# Teaching and evaluation methods of medical ethics in the Saudi public medical colleges: cross-sectional questionnaire study

**DOI:** 10.1186/1472-6920-13-122

**Published:** 2013-09-10

**Authors:** Abdulaziz F AlKabba, Ghaiath MA Hussein, Omar H Kasule, Jamal Jarallah, Mohamed Alrukban, Abdulaziz Alrashid

**Affiliations:** 1 Medical College, Imam Mohammed Bin Saud University, PO Box 59046, 11525 Riyadh, Saudi Arabia; 2 Faculty of Medicine, King Saud bin Abdulaziz University for Health Sciences, PO Box 59046, 11525 Riyadh, Saudi Arabia; 3 King Fahad Medical City, PO Box 59046, 11525 Riyadh, Saudi Arabia; 4 Department of Family & Community Medicine, College of Medicine, King Saud University, P.O. Box: 2925, Riyadh 11461, Saudi Arabia; 5 Medical College, Majmaa University, Riyadh, P.O. Box: 91678, Riyadh 11643, Saudi Arabia; 6 College of Medicine, King Saud University, Riyadh, Saudi Arabia; 7 Pediatric Division, King Abdulaziz University Hospital, P.O. Box: 245, Riyadh, Saudi Arabia

## Abstract

**Background:**

Saudi Arabia is considered one of the most influential Muslim countries being as the host of the two most holy places for Muslims, namely Makkah and Madina. This was reflected in the emphasis on teaching medical ethics in a lecture-based format as a part of the subject of Islamic culture taught to medical students. Over the last few years, both teaching and evaluation of medical ethics have been changing as more Saudi academics received specialized training and qualifications in bioethics from western universities.

**Methods:**

This study aims at studying the current teaching methods and evaluation tools used by the Saudi public medical schools. It is done using a self-administered online questionnaire.

**Results:**

Out of the 14 medical schools that responded, the majority of the responding schools (6; 42.8%), had no ethics departments; but all schools had a curriculum dedicated to medical ethics. These curricula were mostly developed by the faculty staff (12; 85.7%). The most popular teaching method was lecturing (13; 92.8%). The most popular form of student assessment was a paper-based final examination (6; 42.8%) at the end of the course that was allocated 40% or more of the total grade of the ethics course. Six schools (42.8%) allocated 15-30% of the total grade to research.

**Conclusion:**

Although there is a growing interest and commitment in teaching ethics to medical students in Saudi schools; there is lack of standardization in teaching and evaluation methods. There is a need for a national body to provide guidance for the medical schools to harmonize the teaching methods, particularly introducing more interactive and students-engaging methods on the account of passive lecturing.

## Background

### Medical education in Saudi Arabia

The Saudi Arabia is second biggest Arab country with a surface area of 2,150 thousand sq km, and a population of 25.5 million, about 7 million of whom are non-Saudis [1-3]. The Saudi Arabia is the leading Muslim country, being the host for the two most holy sites for Muslims (Mekkah and Medina). Millions, mostly Muslims, come to worship and work every year in different sectors. The most populous regions are those where the biggest cities are located, namely Riyadh, the political capital in the central region; Jeddah, the portal city in the western region; and Dammam in the eastern region.

Compared to other east Mediterranean countries, it has one of the highest gross national income per capita and expenditure on health (USD 22,300 and 6.4% (as percentage of gross domestic product) respectively [2,4].

It has also witnessed an exponential expansion in the higher education institutions in the last five years through the establishment of centers for research and scientific excellence in universities, in addition to assigning more than seven billion Saudi Riyals (about $1.9 billion USD) to send Saudi students in full scholarships in a number of developed countries over the range of five years [5].

According to the government’s data, a total of 636,245 (268,080 male and 368,165 female) students were enrolled in higher education in 2006. Among them, 528,146 students (187,489 male and 340,657 female) were in Bachelor programs, 9,768 students (5,551 male and 4,217 female) were in Master Programs, and 2,410 students (1,293 male and 1,117 female) were in Ph.D. programs. Another 93,968 students (72,199 male and 21,769 female) were in Intermediate Diploma courses and 1,953 students (1,548 male and 405 female) were in Higher Diploma course [4] (http://www.mohe.gov.sa/en/docs/Doc1/VDMPI024.pdf).

The number of universities increased from 15 universities in 2007 to reach 32 as of the end of 2011. The indicator of number of universities per million inhabitants has also increased to 1.2 / million inhabitants in 2010. The most populated areas in the Kingdom have the biggest number of universities. There are 17 universities in Riyadh and Makkah. The total growth rate for institutes and colleges recorded 38%, as their number reached to 434 (39 of which were private) [6].

The indicators of scientific research has also improved over the past five years, as numbers of Masters and PhD graduates increased by 81.9%, and the volume of research in the Kingdom increased by 73.3%, according to ISI classification, and 95% according to Scopus classification. The Kingdom also came in the second rank among Arab States, in terms of research volume [6] and the 25^th^ globally in terms of the quality of its educational system to a competitive economy [7].

This expansion in higher education was also accompanied by an expansion in medical schools. Currently there are 21 well established medical colleges, acknowledged by the Ministry of Higher Education. Four of which are private and 16 of them were established after the year 2001 onwards. Their curricula vary but they are mostly integrated, teacher-based, community-oriented curricula and problem-based learning (PBL) [8]. Most of the medical colleges start their clinical phase in the 4^th^ year in a 6 year college, or the 3^rd^ year in 5-year college. However, all medical schools give their students the chance to visit community-based healthcare centers and to communicate with patients in the first two years.

### Teaching medical ethics globally and in Saudi Arabia

Although teaching medical ethics in medical education can be traced to 2500 years back; teaching it as an academic discipline to medical students is quite new. For instance, in 1972, only 4% of the US medical schools taught medical ethics as a required course, rising to 34% in 1989, and reaching 100% only in 1994 [9]. Similarly in the UK, it was not until 1987 when the Pond Report was issued followed by the influential ‘Tomorrow’s Doctors’ report in 1993 that recommended the inclusion of “ethics and legal issues relevant to the practice of medicine” so that the graduates acheive “an awareness of the moral and ethical responsibilities involved in individual patient care and in the provision of care to populations of patients” that a major shift has taken place in the teaching of medical ethics in the UK [10,11].

The development of the bioethics curricula in the western countries, ahead of many developing countries, was partly explained as a part of the overall western steps ahead in the development of their moral view, the complexity of the healthcare system, and the shift of major causes of death from acute curable diseases to chronic incurable ones [12,13]. These factors have been accompanied by ethical and legal issues that needed to be addressed systematically and consistently through integrating these issues in the medical education.

Along other shifts that took place in the medical education, the teaching methods of medical ethics have gradually moved from the class-based methods with the focus on cognitive pedagogic goals, into more interactive methods that actively engage the students though the use of cases, scenarios, movies and PBL methods.

This shift has resulted from the increasing involvement of scientists and clinicians in teaching medical ethics on the account of the philosophers who were the pioneers in teaching the subject. This is not unique to the western contexts, as a very similar shift has been noticed in teaching ethics in Saudi Arabia. However, the main difference between western and the Saudi shift was in that the leading roles, when it comes to teaching moralities, were given to religious scholars who are not related to the healthcare provision. They tended to give general virtues that the Muslim doctors should hold. They virtues were more general advices and rhetoric, like ‘treat your patients well’, ‘be kind’, ‘altruism’, etc. These were not articulated or operationalized to fit the healthcare delivery model. Moreover, they lacked the provision of a systematic way of defining and resolving the ethical issues that the graduates will encounter as a part of their practice. They obviously lacked the needed level of details to make them usable by the students in their practice. Arguably, this led to some degree of detachment between the ethics that are taught in the lecture rooms and what the doctors face in their practice.

In addition, the field of bioethics shifted from the mere normative philosophical zone to a more empirical, practice-oriented approach. The medical ethics courses in Saudi Arabia, among other Muslim countries, were initially included in the medical curricula as few lectures mainly given by religious scholars, or clinicians with religious sciences background. Gradually, there was a shift from lecture-based teaching to more interactive teaching with scopes more than inculcating the ‘virtuous’ traits that a Muslim doctor should hold. This shift is partly attributed to the shift in the content of the curricula and the teachers who deliver them. To illustrate, religious sciences are usually given in a lecture format where the scholar teaches his students who are only given a chance to ask questions or seek clarifications at the end of the lecture. When ethics was taught by non-provider religious scholars; the teaching format was dominantly lectures. This has gradually changed as more clinicians became trained, or interested, or perhaps forced to teach ethics due to the lack of staff. This shift in the teachers led to more interactive, case-based sessions.

Discussing the content of these curricula was not an objective of this study, but Ypinazar and Margolis have suggested that western ethical reasoning methods may be suitable for teaching medical ethics for Arabic speaking medical students [14]. We do not argue against this conclusion despite the need to have it well-established with further studies related to the contents of the ethics curricula in Saudi Arabia and other Muslim and Arabic-speaking countries. Nevertheless, the novelty of the field led to some level of failure to come up with a common curriculum for the religious aspects of medical practice and the ethics course. Assigned staff had to choose between an approach to teach both the modern form of bioethics and the Islamic virtues in a singly curriculum, or divide the curriculum into literary two curricula: one for the practicalities of ethical issues in clinical practice (under professionalism), and another for the religious aspects.

Again, these approaches did not seem to be perfect, especially with the lack of expertise in teaching medical ethics in its modern format, despite the general agreement on the universality of the main ethical principles that are widely adopted and taught in the western medical schools. Moreover, two of the authors (AA and GH) have argued that the interactive teaching of medical ethics along with other contextual criteria in the medial educational process are needed to achieve the best out of the medical ethics teaching [15].

Despite the amount of studies done on teaching ethics for medical undergraduates globally; studies examining teaching methods in medical ethics education has been identified as a deficit in the literature on medical ethics education [16].

In Saudi Arabia, many studies were done to assess and discuss some aspects related to the medical education in Saudi Arabia [8,17-22]; however, there has been very little available research to study teaching and evaluation methods of medical ethics in the Saudi universities [23,24].

This study aims at helping to fill this gap by studying the methods of teaching and evaluating medical ethics in the Saudi public medical colleges. It does not claim to study the content of these curricula, nor the relation between Islamic ethics and western ethics though this theme is taken into consideration in the discussion of the results.

## Methods

A cross sectional study design was used to survey ethics teaching and evaluation methods used by the governmentally-funded medical colleges in Saudi Arabia. A 100% sample was used covering all public medical schools in Saudi Arabia. The names and addresses of deans of the schools were obtained from the official universities’ directory and an email was sent to each of them explaining the objectives and procedures of the study as well as assurances of confidentiality. Included in the message was an online self-administered questionnaire (http://www.tfaforms.com/233646) that consisted of 5 sections: 1) Demographic Information (region, establishment, and contact information), 2) Information about the institution’s medical ethics curriculum & ethics department/unit (curriculum development and teaching), 3) Medical Ethics Teaching Staff (numbers and qualifications), 4) Teaching Methods, and finally 5) Students’ and course evaluation.

Follow up was made by emails and personal phonecalls to ensure full response. Data from online responses were entered automatically in an MS Excel database and were analyzed using SPSS software version 16. Frequencies with percentages were used for description and the chi square test statistic was used to test for association between variables with the level of significance set at 0.05.

### Ethical considerations

This study was ethically reviewed and approved by the Institutional Review Board at King Fahad Medical City in Riyadh, Saudi Arabia. The confidentiality of the given data was strictly preserved, as only three of the authors had access to the submitted questionnaires. Identifiable data (names and phones) was left optional to the participants to provide for future communications purposes.

## Results

### Study response

Table 1 shows information about medical schools that responded. Fourteen out of 16 (87.5%) medical schools responded. The female and male divisions of the biggest medical school, King Saud University, were treated as 2 separate schools. Each of which has its own curriculum. Their regional distribution corresponded to total population and number of medical schools and was as follows: 4 (28.6%) from Riyadh the most populous province, 3(21.4%) from Makkah the next most populous province and 1(7.1%) from each of the smaller provinces of Tabuk, Sharqiyah, Baha, Qasim, Madina, Najran, and Asir.

**Table 1:** Medical schools that responded

**Province**	**Region**	**Names**	**Number**
Riyadh	Central	King Saud university	4
		Al-Imam Mohammed bin Saud Islamic university	
		King Saud bin Abdulaziz university for health sciences (Riyadh)	
		King Fahd medical city	
Qasim		Al Qassim university	1
Makkah	Western	King Abdulaziz university	3
		King Saud bin Abdulaziz university for health sciences (Mekkah)	
		Taif university	
Madinah		Taibah university	1
Sharqiyah	Eastern	University of Dammam	1
Baha	Southern	Al Baha university	1
Najran		Najran university	1
Asir		King Khalid university	1
Tabuk	Northern	Tabuk university	1

### Ethics departments

The majority of the responding schools, 6 (42.9%), had no ethics departments, 3(21.4%) had a separate ethics department, and 5 (35.7%) taught ethics as part of another department mostly family medicine or community medicine. The earliest 2 departments were established in 1998 and the 2 most recent were established in 2011. One department was established in each of the years: 2001, 2008, and 2010.

### Curriculum development

All schools had a curriculum dedicated to medical ethics. They showed a lot of self reliance in developing curricula; as the faculty staff developed the faculties’ own curricula in 13 of the schools (92.9%). Additional methods of curriculum development included employment of contracted staff by 6 schools (42.9%), adoption from another Saudi medical school by 2 schools (14.3%), and review of the international literature by 4 schools (28.6%).

### Ethics teaching

The majority of schools 13 (92.8%) made learning medical ethics compulsory for students; 5 (35.7%) schools had ethics taught as an independent course while 4 (28.6%) had it taught as part of another course. Ethics was covered in all years of study with some schools teaching it in more than one year of study. The number of schools teaching ethics by year of study was distributed as follows: first year 3, second year 6, third year 7, fourth year 10, fifth year 5, and sixth year 2 medical schools.

### Course credit hours allocated to ethics teaching

The number of credit hours allocated to ethics teaching was limited and varied among schools; 9 schools (64.3%) allocated 2 or less credit hours to the teaching of ethics. Two schools allocated 3 hours, 2 allocated 4 hours, and 1 allocated 5 hours.

### Number and job status of the teaching staff

Twelve schools responded to the question on the number of teaching staff. Six (50.0%) of the schools that responded had 4 or less staff involved in teaching ethics while 6 schools (50.0%) had more than 5 academic staff teaching ethics. One medical school had only one staff teaching ethics. The teaching staff taught ethics in addition to other disciplines. The job status of 47 staff who responded was 41 (87.2%) permanent, 4 (8.5%) visiting, and 2 (4.3%) others.

### Qualifications and experience of the teaching staff

The highest qualifications in ethics reported for 47 staff for whom data was available were as follows: diploma 1 (2.1%), masters 8 (17.0%), short courses 6 (12.8%), and others 20 (42.6%). The countries of qualification for 35 staff with complete information were USA 3 (8.6%), Canada 9 (25.7%), Europe 4(11.4%), Saudi Arabia 10 (28.6%), and other countries 15 (42.9%). Forty one (54.7%) teaching staff responded to the question on years of experience teaching medical ethics. Out of 41 teaching staff who responded about their experience in teaching ethics, the majority of the teaching staff 20 (48.8%) had experience of 4–9 years in teaching ethics, 12 (29.3%) had taught for 10 years or more while 9 (22.0%) had taught for 3 years or less.

### Teaching methods

**Figure 1:**
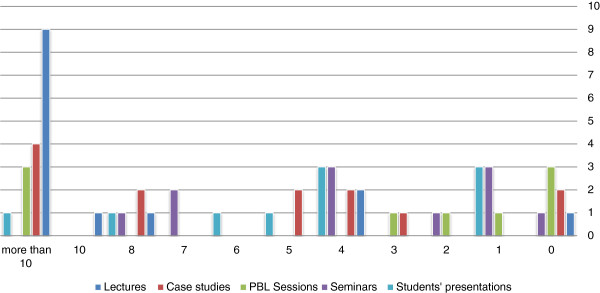
Teaching methods used to teach ethics in Saudi medical colleges.

Figure 1 shows teaching methods reported and the number of sessions per method for the duration of the ethics course. The most popular teaching method was the lecture method used by 9 schools (64.3%) for 10 or more lectures along the course. This was followed in decreasing order of frequency by case studies, PBL sessions, seminars, and student presentations.

### Student assessments

**Figure 2:**
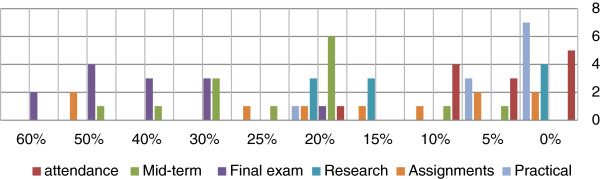
Percentage given to each evaluation method.

**Figure 3:**
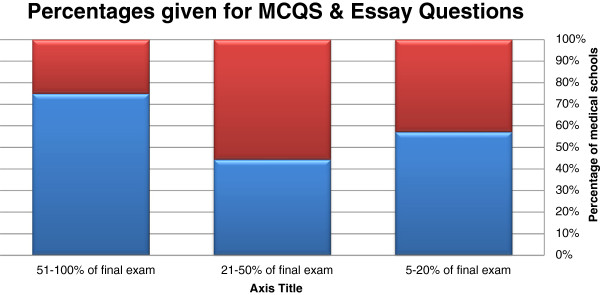
Components of the final assessment.

Figure 2 shows methods of student assessment. The most popular form of student assessment was the final examination that was allocated 40% or more of the total grade by 6 schools (42.9%). Six schools (42.9%) allocated 15-30% of the total grade to research. Eight schools (57.1%) allocated up to 20% of the total grade to attendance. Other assessment methods were insignificant. Figure 3 shows the components of the final assessment. Most schools relied on multiple choice questions followed by essay questions. OSPE and OSCE were not used at all.

### Ethics course evaluation

Eleven (78.6%) schools reported formal evaluation of their courses. Two of the oldest and two of the youngest schools did not report evaluation of their courses.

## Discussion

This is a preliminary study whose objective is to obtain basic information on ethics teaching and evaluation for undergraduate medical students in Saudi Arabia that will be the basis for more detailed studies. The numerators and denominators of many statistics were low being based on 14 medical schools as responding units with an impact on the statistical stability of computed proportions. This was mitigated by the fact that this was a 100% sample that covered all public medical schools in the country.

Although all the responding medical schools teach medical ethics, there are only 3 schools (21.4%) that have a separate department of medical ethics, whereas 5 schools (35.7%) taught ethics as part of another department that was recently established. Though this reflects a growing interest in establishing departments that take care of ethics teaching; it also reflects lack of confidence or experience in figuring out the best way for teaching ethics.

The high proportion of in-house ethics curriculum development is explained by the Islamic background of the country that makes importation of curricula problematic because such curricula may not address legal and moral issues from an Islamic perspective. However, if this is combined with the fact that most of the staff who teach ethics in the Saudi universities are teaching by interest and self-learning; it is possible to conclude that the curricula can have major gaps in terms of the their content. These gaps could be the reason why some medical schools have contracted external experts, relied on other Saudi curricula, or reviewed the international literature, then tried to modify it to the Saudi context.

Teaching ethics is mandatory in almost all public medical schools. This is a good policy which reflects formal acknowledgment of the importance of ethics for medical professionals. That said; there is clear lack of consistency on how and when to teach it. For example, there is a wide variation in the years in which ethics is taught. The variation in the years of teaching ethics to the students reflects a degree of uncertainty on when to teach ethics. Teaching ethics in the first two years of the medical education, i.e. the preclinical phase in most of the Saudi schools, is usually given as a general introduction to the professional attributes that the Muslim doctor should hold and practice. Also, it is worth notifying that most of the medical schools teach medical ethics in the clinical phase (3^rd^-6^th^ years). This is done either as a part of the community and family medicine curricula, or as a separate medical ethics curriculum. Being part of these two curricula specifically can be attributed to two reasons. First, the responsibility of teaching ethics is usually referred to those who teach these two disciples. Secondly, it comes with the trend of putting any ‘non-clinical’ discipline under the umbrella of community medicine. These results need to be seen within the overarching fact that philosophy is never taught in the Saudi schools or universities.

In addition to the common approach to teach ethics in the first year, and in the pre-clinical phase; it is possible that introducing medical ethics in the clinical phase will makes the students appreciate its importance and relevance to their practice. However, it could affect the students’ interest and commitment to the course of medical ethics, of the relatively low credit hours, compared to the major clinical specialties that have double or triple the credit hours given to ethics. These findings are similar to the findings of Moodley when studying teaching ethics in South Africa [25], and the findings by Miyasaka and colleagues who proposed that medical ethics should be taught step by step throughout preclinical and clinical education [12].

Ethics is an emerging discipline in Saudi Arabia being taught in some schools by non-specialists with limited ethics teaching experience and not being housed in designated ethics departments; this situation will change with time. A large proportion of the teaching staff, 28 (37.3%), did not specify if they have obtained any academic qualifications in ethics. Most of these are likely to have taught themselves ethics as a hobby and did not acquire specific or specialized training. This could be thought of as the usual course of events at the start.

There was wide variation among the different schools in the number of credit hours allocated to ethics teaching. This may reflect the extent of interest of teaching staff and curricula committees in different schools. It may also reflect that ethics teaching is still in its infancy and needs more work to convince medical schools of its importance. This will certainly change when academic accreditation (national or international) is sought, where a minimum requirement of credit hours should be allocated for ethics.

In this study, lecturing was found to be the most frequently used method of teaching, which is unfortunate. Ethics is known for its controversial issues and the need to make a support argument and this cannot be learned effectively through lectures. Replacing this by student-centered, PBL methods is urgently needed. However, awaiting that to happen, a mixture of interactive, scenario-based lecture and student presentations can be utilized. McCullough has suggested a skills-development approach that could include (1) exposing students to simulated ethical dilemmas to help students in reconciling the theory they have learned with their intuitive ethics, (2) coupling teaching innovation and development with either formative or summative assessment, and (3) combining communication and ethics strands where possible [26]. Interestingly, we find out that even the medical schools that adopted problem based learning (PBL) curriculum do not provide PBL sessions for teaching ethics per se. It is not even clear if the ethical issues are taught as part of the integrated educational system for other basic or clinical blocks.

The tools of assessment of students in the ethics course were found to be like those used for other courses, i.e. multiple-choice questions (MCQs). The use of MCQ for student assessment is inappropriate for a subject that involves learning concepts as well as methods of reasoning and not factual information. Essays are better because they allow a student to compare and contrast different opinions. From a more practical point of view, the faculty staff found MCQs objective and easier to correct, which is usually done electronically, compared to the ‘human’ load needed to correct essay questions, especially in the schools of relatively high number of students. Moreover, students may find it difficult to express themselves in writing, whether in English (their language of education), or Arabic (their mother tongue) and provide proper arguments.

This is unlike the findings of Mattick and Bligh who found that most of the UK medical schools preferred essays and objective structure clinical examination (OSCE) to other formats of evaluation, including MCQs [10].

Finally, the formal evaluation of ethics teaching by up to 73% of the schools is a good sign that there is motivation to improve, though it is not clear to what extent the feedback they receive is utilized for the improvement of the courses for the next students to come. The current study did not address curriculum content that will be the subject of a subsequent study.

## Conclusions

Teaching of medical ethics in Saudi Arabia is in its initial stages but there are signs that it is along the right track and that the future will be better. The challenges faced by ethics teachers in Saudi Arabia are mainly attributed to lack of guidance on how to develop a uniform curriculum that addresses both the religious aspects and the professional practical aspects that are sourced from western, or other non-Islamic sources in order to help in preparing the graduates to practice in non-Muslim countries, as well as dealing with non-Muslim patients. There is a need for benchmarks established by medical licensing bodies to guide the medical schools in the formulation of their curricula in terms of content, teaching and evaluation methods. Until a critical mass of trained ethicist who can teach medical ethics in the Saudi medical colleges is found, there is a need to train current available teaching staff as ‘training of trainers’ on how to teach medical ethics, regardless their previous qualification in ethics.

## Competing interests

The authors declare that they have no competing interests.

## Authors’ contributions

AB carried out the review of literature, contributed to the development of the data collection methods, contributed to the introduction and conclusion sections. GH carried out the review of literature, developed the online data collection form, communicated with the faculties, and contributed to all the sections. OK contributed to the development of the data collection tools, communications, and wrote the results and the discussion sections. JJ contributed to the review of literature, reviewed the versions of the manuscript and contributed to the final version of the manuscript. MAR contributed to the review of literature, reviewed the versions of the manuscript and contributed to the final version of the manuscript. AA contributed to the review of literature, reviewed the versions of the manuscript and contributed to the final version of the manuscript. All authors read and approved the final manuscript.

## Pre-publication history

The pre-publication history for this paper can be accessed here:

http://www.biomedcentral.com/1472-6920/13/122/prepub
